# Supplementary material for: Campylobacteriosis Agents in Meat Carcasses Collected from Two District Municipalities in the Eastern Cape Province, South Africa
Source: Foods. 2020 Feb 16;9(2):203. doi: 10.3390/foods9020203 (PMC7074574; doi:10.3390/foods9020203)
Supplement: Supplementary file 1 [file foods-09-00203-s001.pdf]

Table S1. Primers sets used for the identification of different *Campylobacter* species.

Target Strain	Primer Sets	Targeted Genes	Base Pairs	PCR Condition (°C)	Cycles	Reference
C. fetus	F: GGTAGCCGCAGCTGCTAAGAT<br>R: AGCCAGTAACGCATATTATAGTAG	*cstA*	359 bp	95°, 95°, 50°, 72°, 72°<br>15', 30'', 1'30'', 1', 7'	25	[41]
C. coli	F: GCTTCGCATAGCTAACAT<br>R: GGTATGATTTCTACAAAGCGAG	*asK*	502 bp	95°, 95°, 50°, 72°, 72°<br>15', 30'', 1'30'', 1', 7'	25	[41]
C. lari	F: TAGAGAGATAGCAAAAGAGA<br>R: TACACATAATAATCCCACCC	*glyA*	251 bp	95°, 95°, 46°, 72°, 72°<br>6', 30'', 30'', 30'' 7'	30	[41]
C. jejuni	F: CAAATAAAGTTAGAGGTAGAATGT<br>R: CCATAAGCACTAGCTAGCTGAT	*cj0414*	161 bp	95°, 95°, 50°, 72°, 72°<br>15', 30'', 1'30'', 1', 7'	25	[41]

Table S2. Oligonucleotide sequence use for detection of virulence genes.

Virulence Factors	Primer Sequence	Targeted Genes	Base Pairs	PCR Conditions	References
Flagella adherence & colonization	F: GGATTTCGTATTAACACAAATGGTGC<br>R: CTGTAGTAATCTTAAAACATTTTG	*flaA*	1723 bp	95°, 94°, 50°, 72°, 72°<br>4', 1', 1', 1', 5'	[24]
C. adherence gene	F: TTGAAGGTAATTTAGATATG<br>R: CTAATACCTAAAGTTGAAAC	*cadF*	400 bp	95°, 94°, 42°, 72°, 72°,<br>4', 1', 1', 1', 5'	[24]
Invasion associated marker	F: GCGCAAAATATTATCACCC<br>R: TTCACGACTACTATGCGG	*iam*	519 bp	95°, 94°, 47°, 72°, 72°<br>4',1', 1', 1', 5'	[42]
C. invasion protein B	F: TTTCCAAATTTAGATGATGC<br>R: GTTCTTTAAATTTTTCATAATGC	*ciaB*	1165 bp	95°, 94°, 43°, 72°, 72°<br>4', 1',1', 1', 5'	[43]
Flagella synthesis & modification	F: GAGCGTTTAGAATGGGTGTG<br>R: GCCAGGAATTGATGGCATAG	*flgR*	390 bp	95°, 94°, 50°, 72°, 72°<br>4', 1',1', 1', 5'	[44]
Cytolethal distending toxin subunit B	F: GTTGGCACTTGGAATTTGCAAGGC<br>R: GTTAAAATCCCCTGCTATCAACCA	*cdtB*	495 bp	95°, 94°, 55°, 72°, 72°<br>4', 1', 1', 1', 5'	[24]

Table S3. Primer sequence use for detection of resistance genes.

Antibiotics	Primer	Primer Sequence (5′–3′)	Base pair	PCR Conditions	Cycle	Reference
Tetracycline	*tetA*	F: GCTACATCCTGCTTGCCTTC<br>R: CATAGATCGCCGTGAAGAGG	201 bp	94°, 94°, 55°, 72°, 72°<br>5', 1', 1', 1.5', 5'	35	[47]
	tetB	F: TTGGTTAGGGGCAAGTTTTG<br>R: GTAATGGGCCAATAACACCG	359 bp	94°, 94°, 55°, 72°, 72°<br>5', 1', 1', 1.5', 5'	35	[47]
	tetC	F: CTTGAGAGCCTTCAACCCAG<br>R: ATGGTCGTCATCTACCTGCC	418 bp	94°, 94°, 55°, 72°, 72°<br>5', 1', 1', 1.5', 5'	35	[47]
	tetD	F: AAACCATTACGGCATTCTGC<br>R: GACCGGATACACCATCCATC	300 bp	94°, 94°, 55°, 72°, 72°<br>5', 1', 1', 1.5', 5'	35	[47]
	tetK	F: GTAGCGACAATAGGTAATAGT<br>R: GTAGTGACAATAAACCTCCTA	460 bp	94°, 94°, 55°, 72°, 72°<br>5', 1', 1', 1.5', 5'	35	[48]
	tetM	F: AGTGGAGCGATTACAGAA<br>R: CATATGTCCTGGCGTGTCTA	158 bp	94°, 94°, 55°, 72°, 72°<br>5', 1', 1', 1.5', 5'	35	[48]

Ciprofloxacin	*gyrA*	F: CGCGTACTATACGCCATGAACCTA<br>R: ACCGTTGATCACTTCGGTCAGG	441 bp	95°, 94°, 55°, 72°, 72°<br>3', 1', 1', 1.5', 5'	35	[49]
Erythromycin	*ermB*	F: CGAGTGAAAAAGTACTCAACC<br>R: GGCGTGTTTCATTGCTTGATG	320 bp	94°, 94°, 55°, 72°, 72°<br>3', 1', 1', 1', 10'	35	[50]
Chloramphenicol	*catI*	F: AGTTGCTCAATGTACCTATAACC<br>R: TTGTAATTCATTAAGCATTCTGCC	320 bp	94°, 94°, 50°, 72°, 72°<br>5', 30'', 30'', 1.5', 5'	30	[51]
	catII	F: ACACTTTGCCCTTTATCGTC<br>R: TGAAAGCCATCACATACTGC	543 bp	94°, 94°, 50°, 72°, 72°<br>5', 30'', 30'', 1.5', 5'	30	[51]
Gentamycin	*aac(3)-IIa (aacC2)*[a]	F: CGGAAGGCAATAACGGAG<br>R: TCGAACAGGTAGCACTGAG	740 bp	94°, 94°, 50°, 72°, 72°<br>5', 30'', 30'', 1.5', 5'	30	[51]
Ampicillin	*ampC*	F: TTCTATCAAMACTGGCARCC<br>R: CCYTTTTATGTACCCAYGA	550 bp	94°, 94°, 45°, 72°, 72°<br>4', 45'', 45'', 45'', 7'	30	[52]
Imipenem	*GES*-1-9<br>*GES*-11	F: AGTCGGCTAGACCGGAAAG<br>R: TTTGTCCGTGCTCAGGAT	399 bp	94°, 94°, 55°, 72°, 72°<br>10', 40'', 40'', 1', 7'	30	[53]
	blas-OXA-48-like	F: GCTTGATCGCCCTCGATT<br>R: GATTTGCTCCGTGGCCGAAA	281 bp	94°, 94°, 55°, 72°, 72°<br>10', 40'', 40'', 1', 7'	30	[53]
	IMP	F: TTGACACTCCATTTACDG<br>R: GATYGAGAATTAAGCCACYCT	139 bp	94°, 94°, 57°, 72°, 72°<br>10', 40'', 40'', 1', 7'	30	[53]
	VIM	F: GATGGTGTTTGGTCGCATA<br>R: CGAATGCGCAGCACCAG	390 bp	94°, 94°, 57°, 72°, 72°<br>10', 40'', 40'', 1', 7'	30	[53]
	KPC	F: CATTCAAGGGCTTTCTTGCTGC<br>R: ACGACGGCATAGTCATTTGC	538 bp	94°, 94°, 57°, 72°, 72°<br>10', 40'', 40'', 1', 7'	30	[53]
